# Chemotherapeutic Drug Based Metal–Organic Particles for Microvesicle‐Mediated Deep Penetration and Programmable pH/NIR/Hypoxia Activated Cancer Photochemotherapy

**DOI:** 10.1002/advs.201700648

**Published:** 2018-01-03

**Authors:** Da Zhang, Ming Wu, Zhixiong Cai, Naishun Liao, Kun Ke, Hongzhi Liu, Ming Li, Gang Liu, Huanghao Yang, Xiaolong Liu, Jingfeng Liu

**Affiliations:** ^1^ The United Innovation of Mengchao Hepatobiliary Technology Key Laboratory of Fujian Province Mengchao Hepatobiliary Hospital of Fujian Medical University Fuzhou 350025 P. R. China; ^2^ The Liver Center of Fujian Province Fujian Medical University Fuzhou 350025 P. R. China; ^3^ Center for Molecular Imaging and Translational Medicine Xiamen University Xiamen 361005 P. R. China; ^4^ The Key Lab of Analysis and Detection Technology for Food Safety of the MOE Fujian Provincial Key Laboratory of Analysis and Detection Technology for Food Safety College of Chemistry Fuzhou University Fuzhou 350002 P. R. China; ^5^ Liver Disease Center The First Affiliated Hospital of Fujian Medical University Fuzhou 350005 P. R. China

**Keywords:** Cu(II)‐drug complexes, hypoxia, metal–organic particles, microvesicle‐mediated deep penetration, programmable activation

## Abstract

A novel metal–organic particle (MOP) based nanodrug formed by mild self‐assembly of chemotherapeutic drugs, including banoxantrone and doxorubicin, through Cu(II)‐mediated coordination effects, is reported. In this nanodrug, Cu(II) acts as a bridge to join AQ4N and DOX, and then, self‐assembly of [‐AQ4N‐Cu(II)‐(DOX)_2_‐Cu(II)‐]*_n_* complexes forms nanosized MOPs (referred to as ADMOPs) through multiple interactions including host–metal–guest coordination, hydrophobic interactions, π‐stacking, and van der Waals force. The ADMOPs reported here have several important features over conventional drugs, including tumor microenvironment pH‐sensitive drug release that can be tracked by “turning on” the fluorescence of AQ4N or DOX through proton competition with Cu(II) to break the coordination bonds and much deeper penetration into solid tumors via microvesicle‐mediated intercellular transfer. Most strikingly, the ADMOPs can serve as stimuli‐responsive nanocarriers to efficiently load the photosensitizer phthalocyanine due to their inherent highly porous characteristics. Thus, the ADMOPs significantly enhance the chemotherapeutic efficacy by “on‐demand” photodynamic therapy, which further induces a hypoxic environment that enhances the reduction of AQ4N to systematically increase the therapeutic efficiency. Taken together, the designed ADMOPs composed of chemotherapeutic drugs may serve as a potential programmable controlled synergistic agent for cancer therapy.

## 1 Introduction

Stimuli‐responsive drug delivery systems (DDSs) hold great promise for overcoming the limitations of conventional chemotherapeutic drugs by allowing “on‐demand” activation of drug effects. Such activation could significantly improve the bioavailability and solubility of drugs, increase the duration of drug efficacy, and reduce side effects by allowing controlled responses to tumor‐specific microenvironment stimuli (such as pH, glutathione (GSH), enzyme, and adenosine triphosphate (ATP), etc.), thus enhancing the antitumoral efficiency.^1^, ^2^ Various stimuli‐responsive DDSs have been designed for cancer treatment, such as pH‐triggered nanocomposites,^3^, ^4^ GSH or temperature‐responsive nanoparticles,^5^, ^6^ and porous‐based smart nanoparticles.^7^, ^8^, ^9^ However, such DDS systems are still unsatisfactory and suffer from various shortcomings, including complicated synthetic processes and low repeatability due to complex designs, limited drug payloads, nonspecific drug leakage and nondegradable compositions.^3^, ^5^, ^9^ Thus, developing a novel stimuli‐responsive DDS with excellent biocompatibility, high drug payload, deep penetration into solid tumors, minimized nonspecific drug activation, and good repeatability with a simple strategy is urgently needed for cancer treatment.

Metal–organic frameworks (MOFs) built simply from metal ions or cluster nodes and organic linkers have been developed using nanotechnology in the past ten years. Some MOF structures that exhibit extremely interesting properties, such as a highly porous structure with pore sizes from 0.4 to 6 nm and a very large surface area, are ideal structures for separation, catalysis, gas‐storage, and exceptional delivery applications.^10^, ^11^ However, most previously published MOF structures (such as Cr‐, Co‐, and Ni‐based MOFs) are not suitable for bioapplications due to their disadvantages including biological toxicity and bioinstability, as hydrolysis or decomposition even can occur in the presence of moisture.^9^, ^10^, ^11^, ^12^, ^13^, ^14^ Recently, nanoscale metal–organic particles, which have been designed for biomedicine applications by judiciously selecting inorganic and organic building blocks, have shown great potential for drug delivery, photodynamic therapy (PDT), photothermal therapy, nucleic acid delivery, and biomedical imaging.^15^, ^16^, ^17^ Therefore, MOPs provide a new direction for developing DDSs. Directly using chemotherapeutic drugs to construct MOPs has exceptional advantages compared with other MOP‐based DDSs, with the highest drug loading efficiency of more than 90%, although such DDSs have not been widely studied.^18^, ^19^

**Figure 1:**
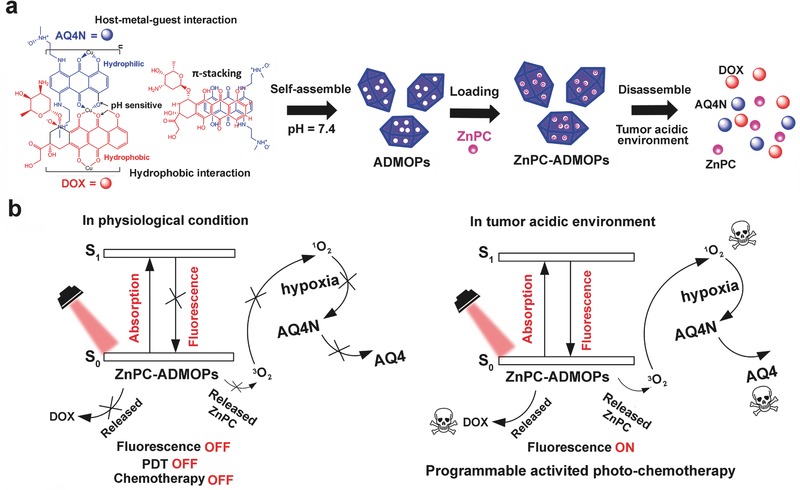
a) Schematic illustration of self‐assembly and disassembly of ADMOPs and ZnPC‐ADMOPs in the low pH condition of tumor microenvironment. b) The programmable activated photochemotherapy mechanisms of ZnPC‐ADMOPs (S_0_, ground state; S_1_, singlet state).

Herein, we report a novel MOP‐based stimuli‐responsive DDS built directly from metal ions and chemotherapeutic drugs that integrates on‐demand PDT therapy to programmably and synergistically treat cancer. The reported MOPs (referred to as ADMOPs) have the inherent characteristics of a highly porous structure for photosensitizer delivery, deep penetration into solid tumors via microvesicle‐mediated intercellular transfer, and precisely programmable response towards tumor microenvironment‐specific pH/hypoxia/NIR for activating cancer photochemotherapy (**Figure** **1**). In our design, the ADMOPs were simply assembled from bioreducible banoxantrone (AQ4N)^20^ and FDA‐approved hydrophobic doxorubicin (DOX) by coordination with Cu(II) ([‐AQ4N‐Cu(II)‐(DOX)_2_‐Cu(II)‐]*_n_* complexes) through host‐metal (C—O—Cu) and metal–ligand (C=O—Cu) multiple interactions, including metal coordination, π‐stacking, van der Waals forces, and hydrophobic interactions. The chemotherapeutic drugs (AQ4N and DOX) inside the ADMOPs were very stable due to fixation via coordination interactions in [‐AQ4N‐Cu(II)‐(DOX)_2_‐Cu(II)‐]*_n_* complexes against drug leakage at physiological conditions, and the fluorescence signals of DOX and AQ4N were also effectively quenched inside the ADMOPs due to self‐aggregation. However, the ADMOPs exhibited low pH‐triggered drug release that could be tracked in real‐time by “turning on” the fluorescence via acidic‐cleavage of coordination bonds between the drugs and Cu(II) ions inside tumors. Furthermore, the ADMOPs could also penetrate solid tumor spheroids much deeper than conventional chemotherapeutic drugs through microvesicle‐mediated intercellular transfer. Importantly, a photosensitizer (phthalocyanine, ZnPC) could be efficiently loaded into the ADMOPs due to their inherent highly porous characteristics, and the photosensitization ability of ZnPC could be well quenched by Cu(II) to decrease the phototoxicity to normal tissues. Furthermore, the photosensitization ability of ZnPC could recover for “on‐demand” PDT inside the tumor since the ADMOPs would be disintegrated by acidic‐based cleavage of coordination bonds; afterward, the PDT‐induced hypoxia could further significantly enhance the chemotherapeutic efficacy by increasing AQ4N reduction to toxic AQ4. Therefore, the reported ADMOPs could serve as a highly promising nanodrug candidate for clinical translation.

## 2 Results and Discussion

### 2.1 Fabrication and Characterization of ADMOPs

**Figure 2:**
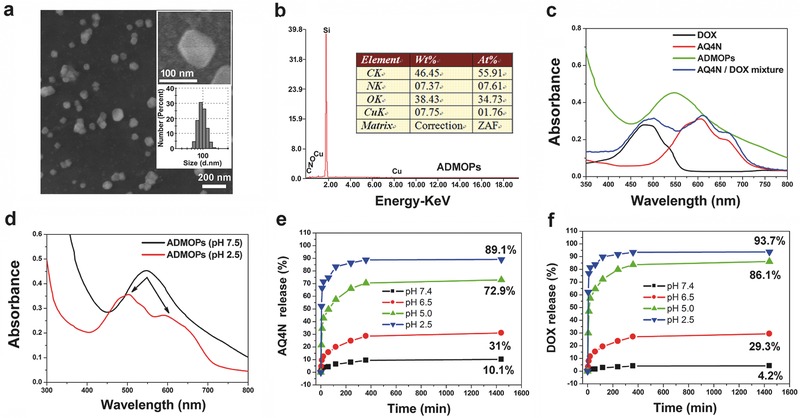
a) Representative SEM images of ADMOPs; the lower inset represents the DLS analysis result of ADMOPs in PBS buffer (pH 7.4). b) Energy dispersive X‐ray spectroscopy of ADMOPs. c) UV–vis–NIR spectra of AQ4N, DOX, AQ4N/DOX mixture, and ADMOPs. d) UV–vis–NIR spectra of ADMOPs in different pH conditions. e) The percentage of AQ4N and f) DOX released from ADMOPs under different pH conditions (2.5–7.4).

The ADMOPs were simply assembled from hydrophilic AQ4N and hydrophobic DOX by coordination between the C—O—Cu and C=O—Cu bonds ([‐AQ4N‐Cu(II)‐(DOX)_2_‐Cu(II)‐]*_n_* complexes). To synthesize the ADMOPs, a mixture containing AQ4N, DOX‐HCl, CuCl_2_ (pH 7.4), and triethylamine (TEA) was first stirred for 24 h, and then added drop‐wise into 10 mL deionized (DI) water. The ADMOPs were finally formed as a bluish violet solution that was obtained after dialysis for 48 h in neutral DI water (Figure 1a). Multiple interactions are crucial for the formation of ADMOPs, including hydrophobic interactions, host–metal–guest coordination, van der Waals forces, and π‐stacking. Scanning electron microscopy (SEM) revealed the formation of nanoparticles, which showed an average diameter of 90 ± 10.1 nm (**Figure** **2**a). The amount of Cu(II) in the ADMOPs was determined by inductively coupled plasma mass spectrometry (ICP‐MS) (see in the Experimental Section). Energy dispersive X‐ray spectroscopy analysis was performed to confirm the elemental composition of the ADMOPs (Figure 2b). In addition, the mass fractions of AQ4N and DOX were obtained from its absorbance values (AQ4N at 600 nm and DOX at 498 nm, respectively) and the corresponding standard curve. The results showed that Cu(II) made up only 9.6 wt%, while AQ4N (26.5 wt%) and DOX (63.9 wt%) assembled into the ADMOPs comprised nearly 90.4 wt% of the loading efficiency, which is tremendously higher than any other drug delivery system thus far;^21^, ^22^ furthermore, the mass fraction composition of each component was consistent with the theoretical values (7.4 wt% of Cu(II), 29.9 wt% of AQ4N, and 67.7 wt% of DOX) according to the molecular structure of [‐AQ4N‐Cu(II)‐(DOX)_2_‐Cu(II)‐]*_n_* with a theoretical molar ratio of 1:2:2 (AQ4N:Cu(II):DOX).

Fourier transform infrared spectroscopy (FTIR) characterization was further conducted to confirm the composition of ADMOPs. As shown in Figure S1 (Supporting Information), the absorption bands at 3387, 3290, 2973, 2910, and 1054.1 cm^−1^ corresponded to the stretching vibrations of the O—H, N—H, —CH_2_, —CH_3_, and C—O groups, respectively. These results prove the existence of DOX and AQ4N in the ADMOPs. Furthermore, the ADMOPs had a weaker absorption band at 1609 cm^−1^ compared with the absorption band of AQ4N and DOX at 1609 cm^−1^, which might be due to the stretching vibration of the C=O group in conjugated ketones by resonance between C—O—Cu and C=O—Cu.^23^ These results prove the existence of Cu(II) in our ADMOPs. The dynamic light scattering (DLS) results showed a relatively narrow size distribution of our obtained ADMOPs, with an average size of 99.3 ± 10.67 nm (Figure 2a, lower inset picture), which was consistent with the SEM results. To study the physiological stability of the ADMOPs, their in vitro release behavior was evaluated in optimal minimal essential medium (opti‐MEM, pH 7.4) with or without 10% fetal bovine serum (FBS) to mimic the in vivo environment. As shown in Figure S2 (Supporting Information), the cumulative release curves showed that the released AQ4N and DOX from ADMOPs were 11.3% and 8.0% in opti‐MEM without FBS within 24 h. While the final released percentage of AQ4N and DOX from the ADMOPs in opti‐MEM with 10% FBS was slightly increased up to 22.4% and 17.8%, respectively, within 24 h, because FBS could bind with Cu(II) to compete with AQ4N and DOX. Even so, the influence of FBS on the stability of our nanodrug was still within an acceptable range. The UV–vis–NIR spectrum was also investigated in vitro. As shown in Figure S3 (Supporting Information), the absorbance spectrum of the ADMOPs in phosphate buffer saline (PBS) (pH 7.4) was only slightly changed after storing for 40 d at 4 °C. These results suggest that our ADMOPs could maintain relatively better structural integrity under physiological conditions. Furthermore, as shown in Figure 2c, compared with the two absorption peaks of the AQ4N/DOX mixture in PBS solution (pH 7.4), the ADMOPs only exhibited one specific absorption peak at 545 nm (red line) at pH 7.4, which was due to the absorbance superposition^24^ However, the single absorption peak of the ADMOPs was divided into two peaks (at 498 and 600 nm) at pH 2.5 (Figure 2d) corresponding to the characteristic peaks of AQ4N and DOX, respectively. This phenomenon suggested that our ADMOPs might be sensitive to low pH to disintegrate the nanostructure, as protons could complete with Cu(II) in acidic conditions.^25^, ^26^

### 2.2 pH‐Sensitive Drug Release and “Turning On” Fluorescence

Inspired by the above assumption, we further carefully investigated the drug release profiles of loaded AQ4N and DOX from ADMOPs in PBS buffer under different pH conditions ranging from 2.5 to 7.4. The amounts of released AQ4N and DOX from the ADMOPs were determined by monitoring the absorbance in the supernatant of PBS solution at different time points. As shown in Figure 2e,f, only 10.1% of the AQ4N and 4.2% of the DOX were released from the ADMOPs after 24 h at pH 7.4 in 37 °C, indicating that our ADMOPs were very stable under physiological pH conditions, while 31% of the AQ4N and 29.3% of the DOX were released from the ADMOPs after 24 h at pH 6.5. More strikingly, the cumulative released percentages of AQ4N and DOX dramatically increased up to 72.9% and 86.1% at pH 5.0 and 89.1% and 93.7% at pH 2.5, respectively, after 24 h of pH change, with extremely rapid release during the first 20 min after changing the pH. SEM and DLS were further used to investigate the structure of the ADMOPs after incubating for 24 h in buffers with different pH values, respectively (Figure S4a,b, Supporting Information). The SEM and DLS results showed that the morphology of the ADMOPs became irregular, and their size became larger in low pH conditions, which may be because the released DOX could interact with itself to form aggregates though π‐stacking after disassembly of the nanodrugs in acidic conditions. To further demonstrate this assumption, the dissociated nanoparticles were centrifuged at low speed, and the supernatant was remeasured by DLS. As shown in Figure S4c (Supporting Information), the supernatant after centrifugation revealed a much smaller particle size at lower pH, while the original ADMOP size distribution with no significant size change was observed at normal pH. These results indicated that the ADMOPs could disintegrate at low pH to release loaded drugs through cleavage of the host–metal coordination bonds in acidic conditions, and therefore, could be applied to respond to the acidic environment of tumors.

**Figure 3:**
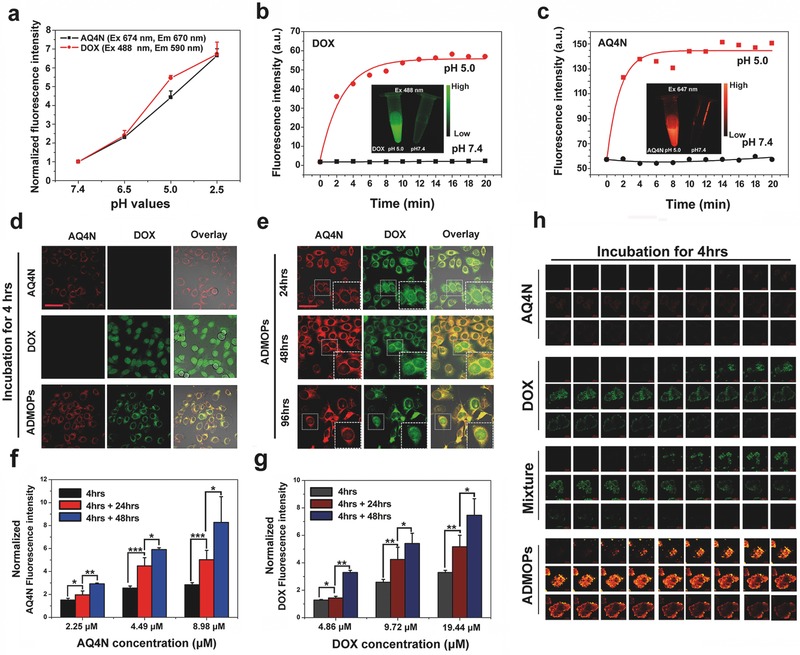
a) Normalized fluorescence intensity of AQ4N and DOX released from ADMOPs under different pH conditions. The fluorescence recovery of b) DOX and c) AQ4N released from ADMOPs under different pH conditions as a function of incubation time; inset: fluorescence images of ADMOPs at different pH values. d) Confocal images of HepG2 cells incubated with free AQ4N, DOX, and ADMOPs for 4 h (scale bar = 50 µm). e) Confocal images of HepG2 cells incubated with ADMOPs for 24, 48, and 96 h. Mean fluorescence intensity of f) AQ4N and g) DOX indicating the controlled release behavior of AQ4N or DOX from the ADMOPs; HepG2 cells were first incubated with ADMOPs for 4 h, then washed with PBS buffer, and subsequently incubated with fresh culture medium for an additional 24 and 48 h (*n* = 4). h) Z‐stack images of 3D tumor spheroids incubated with AQ4N, DOX, AQ4N/DOX mixture, and ADMOPs for 4 h. The images were taken every ≈3.3 mm from the top to the bottom of intact 3D tumor spheroids, scale bar = 100 µm.

Notably, the fluorescence of the ADMOPs was efficiently quenched at pH 7.4 due to the self‐aggregation of DOX and AQ4N, which could be recovered by low pH. As shown in **Figure** **3**a, the fluorescence signals of both AQ4N and DOX from the ADMOPs were increased or recovered with decreasing pH values, and these results were further confirmed by fluorescence imaging (Figure S5, Supporting Information). Furthermore, the fluorescence recovery was very fast and efficient, and the fluorescence signals of both AQ4N and DOX from the ADMOPs quickly increased by several folds within a few minutes when the pH was changed from 7.4 to 5.0 (Figure 3b,c; Figure S6, Supporting Information). These results further clearly confirmed that our ADMOPs could precisely respond to pH change.

### 2.3 Cellular Uptake and Microvesicle‐Mediated Deep Penetration

Next, we evaluated the cellular uptake of the ADMOPs by HepG2 cells via confocal microscopy (CLSM). As shown in Figure S6 (Supporting Information), red fluorescence of AQ4N (excited by 633 nm laser) was readily observed in the cytoplasm of HepG2 cells within the first 30 min of incubation, while green fluorescence of DOX (excited by 488 nm laser) rarely appeared during this time, which might due to the insufficient recovery of DOX fluorescence released from ADMOPs. As the incubation time progressed, both AQ4N fluorescence and DOX fluorescence were concomitantly increased in the cytoplasm of HepG2 cells. To further investigate the uptake efficiency of our ADMOPs, HepG2 cells were treated with free DOX, free AQ4N, and the ADMOPs for 4 h. As shown in Figure 3d, the fluorescence intensity of AQ4N (red) from the ADMOP‐treated cells was much higher than that of free AQ4N‐treated HepG2 cells, indicating that the nanoscale‐sized ADMOPs could be more efficiently uptaken via endocytosis than neutral free AQ4N via diffusion,^27^, ^28^ However, the fluorescence intensity of DOX (green) from the ADMOP‐treated cells was slightly lower than that from the free DOX‐treated cells and exhibited a different cellular distribution pattern compared with free DOX. As shown in Figure 3d,e, the DOX signal from ADMOP‐treated cells was mostly located in lysosomes near the nucleus, while the DOX signal of free DOX‐treated cells was mostly located in the nucleus (Figure 3d). This was not surprising because the ADMOPs should first be delivered to lysosomes to disassociate and then release the DOX. Furthermore, the released DOX after lysosome disassociation in the ADMOP‐treated cells could then slowly move to the nuclei, as indicated by the significantly increased DOX fluorescence in the nucleus along with increased incubation time (for example, after 24, 48, and 96 h of incubation in Figure 3e).

To further confirm the controlled release behaviors of AQ4N or DOX in our ADMOPs, HepG2 cells were first incubated with ADMOPs for 4 h, then extensively washed with PBS buffer, and subsequently incubated with fresh culture medium for an additional 24 and 48 h. The amounts of released AQ4N (excitation at 633 nm, emission at 660 nm) and DOX (excitation at 488 nm, emission at 595 nm) were evaluated by recording the fluorescence intensity of cells in 96‐well plates (*n* = 4). As shown in Figure 3f,g, the fluorescence intensities of both AQ4N and DOX released from the ADMOPs concomitantly increased as the incubation time progressed. These results clearly suggested that our nanosized ADMOPs could be efficiently uptaken by HepG2 cells.

**Figure 4:**
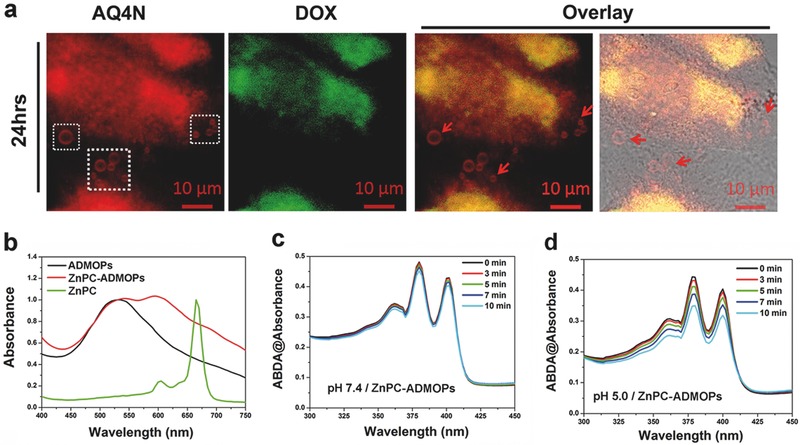
a) Confocal images of HepG2 cells incubated with ADMOPs for 24 h. b) UV–vis–NIR spectra of ADMOPs and ZnPC‐ADMOPs in PBS buffer and ZnPC in DMSO. c,d) The absorbance of 9,10‐dimethylanthracene (ABDA, 100 × 10^−6^ m) after photodecomposition by ROS generation upon 670 nm laser irradiation at 0.1 W cm^−2^ in the presence of ZnPC‐ADMOPs for different times at pH 7.4 and 5.0.

Tumors are characterized by unique microenvironments to protect and promote cancer cell progression, which include upregulated glycolysis to lower the extracellular pH in tumors and facilitate the invasion and metastasis of cancer cells.^29^, ^30^ As indicated by our very convincing results presented above, the ADMOPs could very precisely release the payload drugs inside tumors while minimizing nonspecific drug release in normal tissues, thus significantly decreasing systematic toxicity and increasing therapeutic efficiency. In addition to low pH, tumors generally have very dense mass structures that cause poor penetration of conventional chemotherapeutic drugs and a hypoxic environment that can lower the therapeutic efficiency of drugs and promote cancer cell metastasis. Increasing the penetration depth of chemotherapeutic drugs is crucial for improving the therapeutic efficiency. We discovered that our ADMOPs could reach the inner side of tumor spheroids and penetrate much deeper than both chemotherapeutic drugs AQ4N and DOX alone. In this study, we chose a HepG2 tumor‐spheroid model to mimic the 3D environment in vivo to evaluate the penetration behavior of the ADMOPs. HepG2 tumor spheroids were treated with free AQ4N, free DOX, a AQ4N/DOX mixture, and the ADMOPs for 4 and 24 h. As shown in Figure 3h and Figure S8 (Supporting Information), AQ4N was primarily immobilized in the outer cell layer, and only few AQ4N particles entered the center of the free AQ4N‐treated spheroids; similar results were obtained for free DOX‐ and AQ4N/DOX mixture‐treated tumor spheroids under the same conditions. However, substantial migration of AQ4N from the periphery to the deep center of the tumor spheroids was observed in the ADMOP‐treated spheroids. As shown in Figure 3h, the fluorescence of AQ4N in the deep center of the ADMOP‐treated spheroids was much higher than that of the free AQ4N‐ and AQ4N/DOX mixture‐treated spheroids. Surprisingly, the above results showed that the ADMOPs could deeply penetrate to the center of densely packed tumors. To further study the mechanisms of ADMOP‐mediated deep penetration, we used CLSM to investigate the interactions between ADMOPs and HepG2 cells. As shown in **Figure** **4**a, we discovered that several AQ4N‐loaded micron‐grade endogenous particles (microvesicles, MVs) appeared in ADMOP‐treated HepG2 cells, and MV transmission among neighboring cells through cell interactions and exocytosis was detected after incubation for 24 h (indicated by white gridlines). Furthermore, only red fluorescence of AQ4N was detected in such MVs. According to a previous study, AQ4N primarily localizes to the endoplasmic reticulum (ER) membrane under aerobic conditions^29^ while DOX is primarily embedded in DNA in the nucleus to inhibit cell proliferation rather than in the ER membrane like most hydrophobic drugs.^30^ As MVs are mainly derived from the ER membrane in cells, it is not surprising that only AQ4N but not DOX was efficiently secreted via the innate biological transport system of MVs. To further confirm the MV‐mediated intercellular transfer of AQ4N, HepG2 cells were treated with ADMOPs for 4 h to allow internalization and then washed with PBS buffer three times to completely remove free ADMOPs. Next, the cells were further incubated in fresh culture medium for another 24 h to allow secretion of AQ4N‐containing vesicles. Subsequently, the culture supernatant was collected and added to untreated HepG2 cells, and these cells were further incubated for 4 h. Finally, CLSM was used to analyze possible vesicle transfer. Any vesicle‐mediated drug transfer would be able to be detected by the corresponding fluorescence signals in the supernatant‐treated cells. As expected, only red fluorescence of AQ4N but not green fluorescence of DOX could be observed in the cytoplasm of the supernatant‐treated cells (Figure S9, Supporting Information). Taken together, these results clearly suggested that our ADMOPs could guide the deep penetration of certain chemotherapeutic drugs across tumor barriers to improve the therapeutic efficiency.

As our ADMOPs could be efficiently uptaken by cancer cells and penetrate deep into the center of tumor spheroids, we next assessed the synergistic chemotherapy outcomes of ADMOPs by using a CCK8 assay. Liver cancer cells (HepG2) were treated with free DOX, free AQ4N, Cu(II) ions or the ADMOPs for 48 h. As shown in Figure S10 (Supporting Information), free DOX alone (22.52 µg mL^−1^) induced relatively high toxicity in HepG2 cells (with 35.2% of cell viability), which was consistent with reports in the literature. In contrast, the viability of cells treated with free AQ4N alone (9.36 µg mL^−1^) was as high as 94%, which might due to the lack of toxicity of AQ4N in aerobic conditions.^27^, ^31^ Interestingly, Cu(II) alone also exerted a certain degree of cytotoxicity (with 69.2% of cell viability), which was lower than free DOX but higher than free AQ4N. This result was also consistent with previously reported studies regarding the anticancer properties of copper complexes.^32^ However, the ADMOPs had significantly higher cytotoxicity (with 21.9% of cell viability) than free DOX, free Cu(II), and free AQ4N due to the higher uptake of the nanosized form and the synergistic anticancer effects of DOX, Cu(II), and AQ4N.

### 2.4 Characterization of the ZnPC‐Loaded ADMOPs

Previous evidence has suggested that the reduction of AQ4N to toxic AQ4 could be enhanced by increasing the local hypoxic level of tumors to further increase cancer cell killing.^27^, ^33^ Inspired by this possibility, we attempted to load the ADMOPs, which has inherent porous characteristics and can dynamically respond to specific tumor environments, with a hydrophobic photosensitizer (ZnPC). In this way, we aimed to enhance the local hypoxia of the tumors by on‐demand PDT treatment to further increase the chemotherapy efficiency under NIR laser irradiation, thus ultimately improving the synergistic therapeutic effects. First, we performed a “N_2_ adsorption–desorption isotherm” experiment to evaluate the pore size of the ADMOPs and the possibility of ZnPC incorporation in the nanodrug. As shown in Figure S11a (Supporting Information), the ADMOPs showed a porous structure with a pore size of 1.67 nm, which is large enough for ZnPC loading (76.2 A^2^). Furthermore, the UV–vis–NIR spectrum and the fluorescence spectrum were analyzed to confirm the successful loading of ZnPC into the ADMOPs (ZnPC‐ADMOPs). As shown in Figure 4b, the as‐prepared ZnPC‐ADMOPs exhibited a new absorption peak at 675 nm corresponding to the characteristic absorption peak of ZnPC, which was not present in the original ADMOPs in PBS buffer (pH 7.4). In addition, the fluorescence intensity of ZnPC in the ZnPC‐ADMOPs was almost completely quenched by Cu(II) inside the ADMOPs compared with the same amount of free ZnPC in dimethylsulfoxide (DMSO) (Figure S12a, Supporting Information).^34^ Interestingly, the fluorescence intensity of ZnPC in the ZnPC‐ADMOPs could easily recover in low pH conditions due to disintegration of the nanoparticles (Figure S12a, Supporting Information). Furthermore, the elemental composition of the ZnPC‐ADMOPs was also analyzed by energy dispersive X‐ray spectroscopy (EDS) (Figure S12b, Supporting Information).

The reactive oxygen species (ROS) generation ability of the ZnPC‐ADMOPs was further investigated using a ROS indicator (9,10‐anthracenediyl‐*bis*‐(methylene) dimalonic acid, ABDA).^35^, ^36^ Interestingly, the ZnPC‐ADMOPs had almost no detectable impact on ABDA absorbance under 670 nm laser irradiation (0.1 W cm^−2^) at pH 7.4, even with irradiation for 10 min, because the ROS generation ability of ZnPC was quenched by Cu(II) inside the intact ADMOPs at pH 7.4 (Figure 4c,d).^34^ However, a sharp time‐dependent decline in ABDA absorbance was observed when the ZnPC‐ADMOPs were preincubated at pH 5.0 for 20 min and then irradiated with NIR laser. This phenomenon was due to the acidic‐triggered ADMOP disintegration, making ZnPC freely available and allowing recovery of the ROS generation ability from the metal‐ion‐based quenching. Therefore, the ZnPC‐ADMOPs could be used for synergistic “on‐demand” PDT and chemotherapy for tumor treatment.

### 2.5 Cytotoxicity in PDT‐Induced Hypoxia

**Figure 5:**
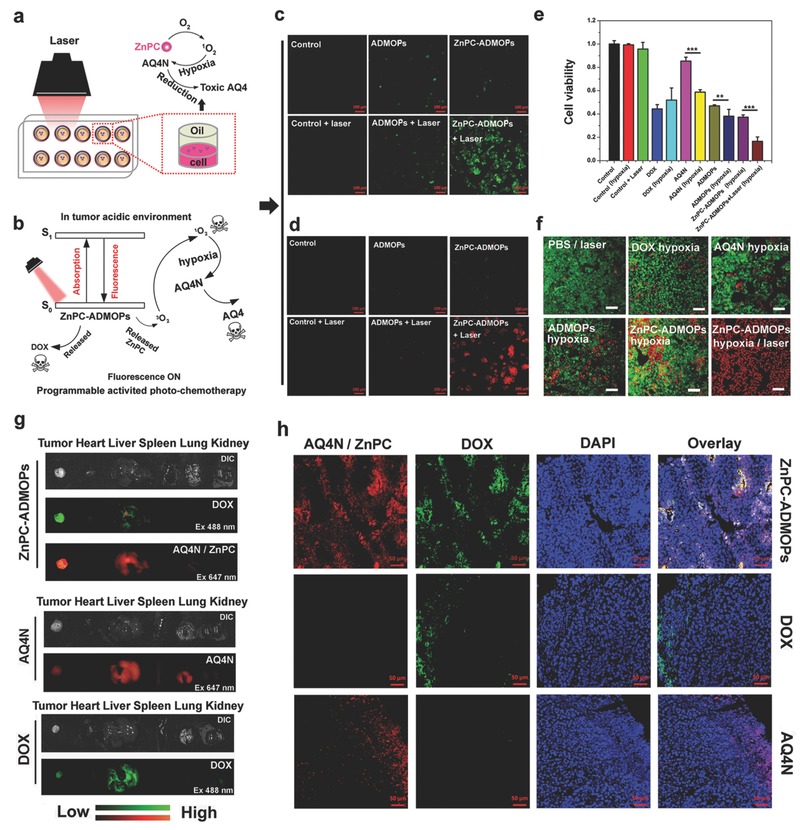
a) Schematic view of the preparation of hypoxic environment in the tumor cell culture under 670 nm laser irradiation. b) The programmable activated photochemotherapy mechanism of ZnPC‐ADMOPs in cancer cells. c) Confocal images of HepG2 cells that received different treatments as indicated; DCFH‐DA was used as a ROS indicator. d) PDT‐induced hypoxic environment in HepG2 cells under different treatments, as indicated. e) Cell viability of HepG2 cells treated with free DOX, AQ4N, ADMOPs, and ZnPC‐ADMOPs with or without laser irradiation (*n* = 3) under the hypoxic environment; the statistical analysis was performed with two‐tailed paired Student's *t*‐tests (*p < 0.05, **p < 0.01, ***p < 0.001). f) Fluorescence images of the live/dead viability/toxicity kit‐stained HepG2 cells under the different treatments, as indicated, scale bar = 50 µm. g) Ex vivo images of tumors and other organs 4 h after intravenous injection of free AQ4N, free DOX, and ZnPC‐ADMOPs (AQ4N or ZnPC, excitation 647 nm; DOX, excitation 488 nm). h) Confocal images of tumor sections; ZnPC/AQ4N are shown in red, DOX is shown in green, and nuclei are shown in blue (DAPI).

Next, we examined PDT‐induced local hypoxia, as follows: HepG2 cells were first incubated with ZnPC‐ADMOPs for 4 h and washed three times with PBS buffer at room temperature. Then, 50 µL fresh medium containing the ROS fluorescence indicator (2,7‐dichlorofluorescein diacetate, DCFH‐DA) was added to the 96‐well plates, followed by adding 100 µL oil (nontoxic) to the upper surface of the medium (**Figure** **5**a,b).^27^ Under 670 nm laser irradiation, the ZnPC‐ADMOP‐treated HepG2 cells exhibited significantly stronger green fluorescence compared with ZnPC‐ADMOP‐ and ADMOP‐treated cells without laser irradiation or untreated HepG2 control cells (Figure 5c). These results clearly proved that the ZnPC‐ADMOPs could selectively produce ROS upon laser irradiation. To further confirm that the PDT selectively induced local hypoxia in HepG2 cells, which is crucial for enhancing the chemotherapy efficiency of AQ4N, a hypoxia detection probe (MitoXpress Kit) was used.^27^, ^28^ As shown in Figure 5d, much stronger fluorescence (red color) was observed by CLSM in ZnPC‐ADMOP‐treated HepG2 cells upon laser irradiation, indicating a high level of PDT‐induced hypoxia in the environment. In contrast, there was no obvious red fluorescence in the ZnPC‐ADMOP‐treated HepG2 cells without laser irradiation. Furthermore, a CCK8 assay was also performed to evaluate the synergistic therapeutic efficiency of the ZnPC‐ADMOPs, which could integrate PDT treatment and PDT‐induced hypoxia‐enhanced chemotherapy against tumor cells. As shown in Figure 5e, AQ4N induced greater inhibition of HepG2 cell proliferation in hypoxic conditions (41.3% inhibition) than in aerobic conditions (14.7% inhibition). Similarly, the ADMOPs inhibited 61.9% of cell proliferation in hypoxic conditions, corresponding to greater inhibition of cell proliferation than in aerobic conditions (53.1%). In contrast, DOX‐treated HepG2 cells exhibited slightly lower inhibition of cell proliferation in hypoxic conditions (48.1%) than in aerobic conditions (55.7%), which might be due to hypoxia‐mediated drug resistance. Notably, the proliferation of cells treated with ZnPC‐ADMOPs and then irradiated by NIR laser was inhibited by 83.5%, which was significantly greater than inhibition of cells treated with ZnPC‐ADMOPs but without laser irradiation (62.7%) in hypoxia conditions. Two key reasons for the enhanced cancer cell killing effect are as follows: First, the combination of PDT itself could increase the cell killing, and second, the PDT‐induced hypoxic environment could further increase the cell killing efficiency of AQ4N. Moreover, a live/dead viability/cytotoxicity assay was also performed to confirm the above observations. As shown in Figure 5f and Figure S13 (Supporting Information), HepG2 cells treated with free AQ4N showed vivid green fluorescence throughout, indicating the low cytotoxicity of AQ4N in aerobic conditions. However, several cells with red fluorescence could be clearly observed among AQ4N‐treated HepG2 cells in hypoxic conditions due to the reduction of AQ4N to toxic AQ4. A similar phenomenon was also observed in ADMOP‐treated cells. Most significantly, many dead cells were observed among ZnPC‐ADMOP‐treated HepG2 cells under PDT‐induced hypoxic conditions. To further investigate the apoptosis mechanisms of ZnPC‐ADMOP‐treated HepG2 cells with laser irradiation, a western blot analysis was performed. As shown in Figure S14 (Supporting Information), the up‐regulation of Bax and the simultaneous down‐regulation of Bcl‐2 were detected in HepG2 cells treated with ZnPC‐ADMOPs under laser irradiation, and these expression changes were more obvious than in cells treated with free AQ4N, free DOX or ADMOPs. The above results clearly suggested that the ZnPC‐ADMOPs acted as a programmable synergistic PDT/chemotherapy agent for cancer treatment.

### 2.6 Antitumor Efficiency In Vitro and In Vivo

To ensure the synergistic antitumoral efficiency in vivo, we first investigated the biodistribution of the ZnPC‐ADMOPs in HepG2 tumor‐bearing mice after intravenous injection. As shown in Figure S15a (Supporting Information), the biodistribution profiles, which were analyzed through the concentrations of Cu/Zn determined by ICP‐MS or through the fluorescence intensity of AQ4N/DOX determined by fluorescence imaging of the main organs, showed that the ZnPC‐ADMOPs mainly accumulated in the liver, tumor, kidneys, lungs, heart, and spleen 4 h after injection. Compared with the results determined by ICP‐MS, the tumor accumulation of ZnPC‐ADMOPs seemed overestimated by the fluorescence imaging, as the complex is likely to disassociate from tumor tissues by design and then produce stronger fluorescence. Although accumulation in the tumor was not the highest, the tumor was still one of the primary regions of nanodrug accumulation determined by ICP‐MS. Furthermore, to ensure the enhanced tumor accumulation of the nanodrug compared with free drugs, animals were sacrificed 4 h after intravenous injection of free AQ4N, free DOX or ZnPC‐ADMOPs. As shown in Figure 5g, compared with free AQ4N‐ and free DOX‐treated mice as controls, much stronger AQ4N/ZnPC (red) and DOX (green) fluorescence signals could be clearly observed inside the tumors of ZnPC‐ADMOP‐treated mice 4 h after intravenous injection, indicating better tumor accumulation of the nanosized drug. Free AQ4N and free DOX were mainly accumulated in the liver and lungs rather than in the tumor tissues, which was consistent with previous studies.^37^, ^38^, ^39^ However, it may be necessary to prolong the observation time to investigate the full drug metabolic process and the ultimate organ distribution in future studies. Furthermore, an ex vivo fluorescence analysis of the excised tumor tissues was also performed to confirm these results. As shown in Figure 5h, AQ4N/ZnPC (red) and DOX (green) fluorescence in the ZnPC‐ADMOP‐treated mice was found in the centers of the tumors, while fluorescence in free DOX‐ and free AQ4N‐treated mice was only observed at the edges of the tumors, indicating deeper and better penetration of the ZnPC‐ADMOPs into tumor tissues. Taken together, these data clearly confirmed that the ZnPC‐ADMOPs could be selectively internalized into the tumor center with excellent deep penetration for cancer therapy.

**Figure 6:**
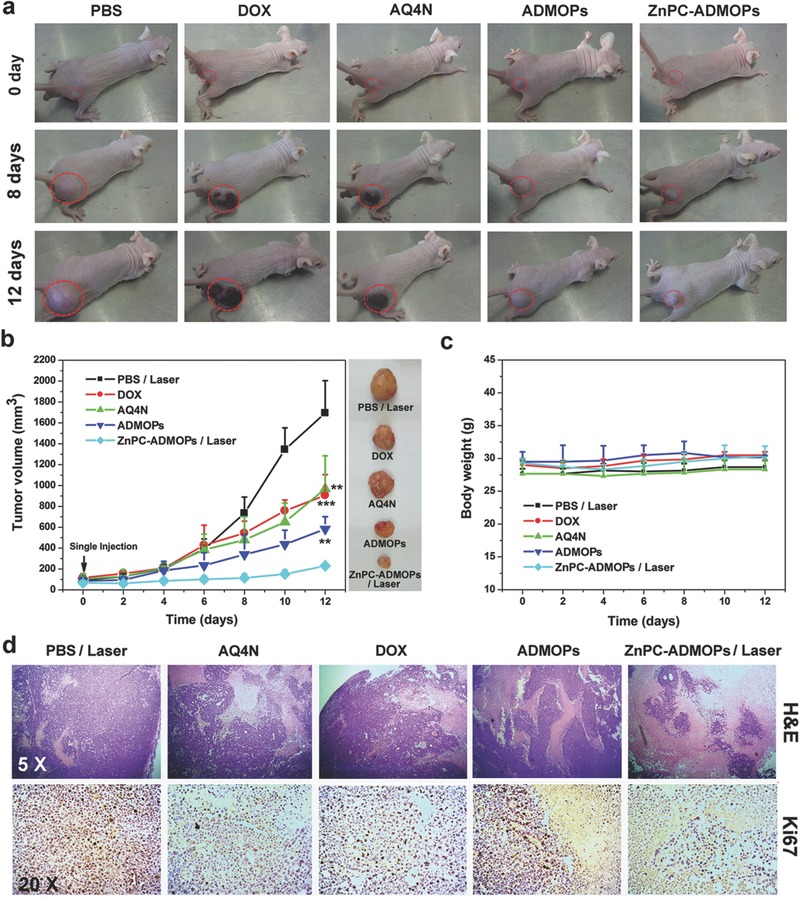
a) In vivo therapeutic response to PBS with laser irradiation, AQ4N alone, DOX alone, ADMOPs, and ZnPC‐ADMOPs with the 670 nm laser irradiation 4 h after intravenous injection (0.1 W cm^−2^, 10 min). b) Tumor volumes of mice after different treatments; all data are presented as the mean ± SD (*n* = 4), and the statistical analysis was performed with two‐tailed paired Student's *t*‐tests (**p < 0.01, ***p < 0.001). c) Mean body weights of mice after different treatments. d) Representative images of H&E and Ki67 staining from tumor sections after the indicated treatments.

Inspired by the high therapeutic efficiency of the ZnPC‐ADMOPs in vitro and the extensive tumor accumulation and deep tumor penetration in vivo, we then carefully evaluated the in vivo synergistic antitumoral efficacy of the ZnPC‐ADMOPs in HepG2 tumor‐bearing mice. As shown in **Figure** **6**a,b and Figure S16 (Supporting Information), tumor‐bearing mice treated with PBS followed by laser irradiation showed rapid tumor growth, while mice treated with AQ4N or DOX alone exhibited a certain delay in tumor growth and modest therapeutic effects. ADMOP‐treated mice exhibited a stronger delay in tumor growth and greater therapeutic efficiency due to the combined therapeutic effects of both AQ4N (for the inner hypoxic tumor cells) and DOX (for the aerobic tumor cells) released from the ADMOPs via tumor microenvironment‐specific pH‐triggered drug release inside the solid tumors. Strikingly, the ZnPC‐ADMOP‐treated mice with laser irradiation had excellent and much stronger tumor growth inhibition compared with all other groups due to the synergistic therapeutic effects of “on‐demand” PDT, cell death pathway activation by PDT‐generated ROS, and extreme hypoxia induced by simultaneous O_2_ consumption of PDT to enhance AQ4N reduction to toxic AQ4. These results were further confirmed by immunohistochemical, H&E, and Ki67 staining of tumors after treatment. As shown in Figure S17 (Supporting Information), compared with the ZnPC‐ADMOP‐treated mice without laser irradiation, much more extensive hypoxic areas were observed in ZnPC‐ADMOP‐treated mice under 670 nm laser irradiation due to the PDT‐induced hypoxia. As shown in Figure 6d, the ZnPC‐ADMOP‐treated mice with NIR laser irradiation exhibited much higher therapeutic efficiency than any other group. Moreover, the systematic low toxicity of the nanodrug is crucial for its clinical translation. High drug toxicity generally leads to severe weight loss; however, we discovered that the mice injected with ZnPC‐ADMOPs followed by PDT treatment had no significant body weight loss (Figure 6c), which might suggest the low systematic toxicity of our nanodrug. These results were further confirmed by pathological assessments. As shown in Figure S18 (Supporting Information), there was no noticeable tissue damage in any major organ compared with the control group (PBS). Together, these results clearly demonstrated that our designed ZnPC‐ADMOPs exhibited excellent synergistic antitumoral efficacy with minimal side effects.

## 3 Conclusion

In summary, we directly applied chemotherapeutic drugs and metal ions to construct a MOP structure (ADMOPs). We report that the MOP structure has advantages of extremely high drug payload (higher than 90% and higher than any previously reported DDS), tumor microenvironment‐specific pH‐responsive ability, and excellent deep penetration ability into tumor tissues. After efficiently loading a photosensitizer (ZnPC) into the pores of ADMOPs (named as ZnPC‐ADMOPs) to form the final nanodrug, the ZnPC‐ADMOPs acted as a smart programmable synergistic nanotherapeutic platform for cancer treatment by combining the “on‐demand” PDT effect, the chemotherapy effect, and the PDT‐induced hypoxic effect to systematically enhance the toxicity of chemotherapeutic drugs. The significantly improved synergistic therapeutic effects of our ZnPC‐ADMOPs were proven both in vitro and in vivo, with minimal side effects. Therefore, the ZnPC‐ADMOP nanodrug reported herein may be a promising strategy to improve the therapeutic efficiency against cancer for clinical translation.

## 4 Experimental Section

*Materials*: Metal phthalocyanine, DOX, AQ4N, DCFH‐DA, and ABDA were purchased from Sigma‐Aldrich. A MitoXpress Kit was purchased from Cayman Chemical Company. Sodium citrate tribasic dehydrates and copper chloride dihydrate were purchased from J&K Scientific. DI water with a resistivity of 18.2 M Ωcm was obtained from a Milli‐Q Gradient System (Millipore, Bedford, MA, U.S.) and was used for all experiments. Unless specified, all other chemicals were commercially available and used as received.

*Cell Culture*: HepG2 cells (hepatocellular carcinoma) were purchased from ATCC (Manassas, VA). All cells were cultured in RPMI 1640 medium (ATCC, Manassas, VA) supplemented with 10% FBS and 100 IU mL^−1^ penicillin–streptomycin (Cellgro, Manassas, VA).

*Preparation of ADMOPs*: First, 1 mg mL^−1^ DOX‐HCl and 1 mg mL^−1^ AQ4N were mixed in DI water, and then, 0.1 mg mL^−1^ CuCl_2_ (pH 7.4) was added to the mixture and stirred for 4 h. Afterward, 450 µL TEA was added to the above mixture by vigorously stirring overnight in the dark at room temperature. The mixture was then added drop‐wise to 10 mL DI water by vigorously stirring. The free AQ4N, DOX, and Cu(II) ions were removed by dialysis for 2 d, changing the DI water every 4 h. The amount of Cu(II) bound to ADMOPs was determined to be 8.518 µg mL^−1^ by XSERIES 2 ICP‐MS (Thermo, USA). The amounts of both AQ4N and DOX in the ADMOPs were determined by measuring the corresponding absorbance at 498 nm for DOX and 600 nm for AQ4N after incubation with PBS buffer at pH 2.5 for 48 h. Briefly, the absorbance of AQ4N was directly measured at 600 nm, as DOX has negligible absorption at this wavelength, and the amount of AQ4N was then calculated from the standard curve, which has a very good linear relation with AQ4N for concentrations of 1–50 µg mL^−1^ (*Y* = 0.005*x* + 0.033, *R* ^2^ = 0.998). Afterward, the corresponding absorbance value of the same amount of AQ4N at 498 nm was calculated from its corresponding standard curve. Then, the corresponding absorbance value of DOX at 498 nm was calculated by subtracting the absorbance value of AQ4N from the total absorbance at this wavelength. The amount of DOX was calculated from its standard curve, which has a linear relation with DOX for concentrations of 1–50 µg mL^−1^ (*Y* = 0.003*x* + 0.031, *R* ^2^ = 0.995). Finally, the AQ4N and DOX concentrations in the ADMOPs (88.2 µg mL^−1^) were determined to be 23.4 and 56.3 µg mL^−1^, respectively. Finally, the mass fraction of each component was calculated as follows: wt% = (weight of interest/weight of ADMOPs) × 100%. The theoretical values of the mass fraction of each component in the ADMOPs were calculated by the following formula: wt% = (molecular weight of interest/total molecular weight) × 100%, based on the molecular structure of [‐AQ4N‐Cu(II)‐(DOX)_2_‐Cu(II)‐]*_n_* with a theoretical molar ratio of 1:2:2 (AQ4N: Cu(II):DOX).

*Preparation of ZnPc‐ADMOPs*: First, the ADMOPs were collected by centrifugation (13 000 rpm min^−1^) and resuspended in PBS buffer (pH 7.4). Then, 1 mg mL^−1^ ZnPC (dissolved in DMSO) was added drop‐wise and further stirred for 24 h. Any unloaded ZnPC was precipitated by allowing the mixture to stand for 12 h. The supernatant was collected, and the precipitate was then dissolved in DMSO. The standard curve had a very good linear relation with ZnPC in DMSO for concentrations of 3.125–50 × 10^−6^ m (*Y* = 0.023*x* + 0.149, *R* ^2^ = 0.995) (Figure S11b, Supporting Information). The concentrations of AQ4N, DOX, and ZnPC in the ZnPC‐ADMOPs (116.9 µg mL^−1^) were determined to be 23.4, 56.3, and 28.6 µg mL^−1^, respectively.

*pH‐Sensitive Fluorescence Spectral Analysis of ADMOPs*: First, 0.2 mg ADMOPs was dissolved in 1 mL PBS buffer with different pH, as indicated (pH 2.5, 5.0, 6.5 or 7.4), and incubated for different times. The fluorescence spectra were then recorded in a quartz cuvette using an Agilent Cary Eclipse fluorescence spectrophotometer (Santa Clara, USA). The excitation wavelengths were 488 nm or 633 nm, and the emission wavelengths were in the range of 450–600 nm or 625–750 nm with both excitation and emission slits of 10 nm under a PMT voltage of 950 V.

*pH‐Triggered Release of AQ4N or DOX from ADMOPs*: The pH‐sensitive release study was conducted as follows: 0.2 mg ADMOPs was dispersed in 1 mL PBS buffer with different pH values, as indicated (pH 2.5, 5.0, 6.5 or 7.4). At predetermined time intervals, 0.5 mL of the supernatant was analyzed to determine the amount of released drug by the UV−vis absorption spectrum after centrifugation at 13 000 rpm min^−1^ for 10 min. To maintain a constant volume, 0.5 mL PBS buffer with the corresponding pH was added after each sampling.

*Confocal Fluorescence Microscopy of the Cellular Uptake of ADMOPs*: The cellular uptake of ADMOPs by HepG2 cells was investigated by using CLSM. In a typical experiment, HepG2 cells (5 × 10^4^) were seeded onto 35 mm glass‐bottom Petri dishes and cultured for 24 h at 37 °C in an incubator. The ADMOPs were added to the cells, and the cells were further incubated for 4, 24, 48, and 96 h in aerobic conditions. The cells were then washed three times with PBS (pH 7.4) at room temperature and fixed with 4% paraformaldehyde for 15 min. Finally, the cells were imaged by a confocal microscope (Carl Zeiss LSM 780, Germany) with 488 nm laser excitation for DOX and 633 nm laser excitation for AQ4N.

*For the 3D Tumor Sphere Assay*: HepG2 cells were seeded as single‐cell suspensions at a concentration of 10 × 10^4^ cells per well in 6‐well ultralow attachment plates (Corning, Corning, NY, USA) in complete medium. After 12 d, the cells were centrifuged at 300 g for 5 min, and the tumor spheroids ≈300 µm were counted. The spheroids were incubated with DOX, AQ4N, AQ4N/DOX mixture or ADMOPs for 4 and 24 h, and then washed with PBS buffer at room temperature. Finally, the tumor spheroids were imaged by CLSM.

*Synergistic Antitumor Efficacy of ADMOPs and ZnPC‐ADMOPs In Vitro*: A cell counting kit (CCK8) was used to study the synergistic antitumor effects of ADMOPs and ZnPC‐ADMOPs against HepG2 cells. Briefly, cells were seeded in a 96‐well plate at a density of 1 × 10^4^ cells per well and incubated in a humid atmosphere (with 5% CO_2_) for 24 h. Then, the original cell culture medium was discarded, and the cells were washed three times with PBS to remove dead cells, followed by incubation with free DOX, free AQ4N, ADMOPs or ZnPC‐ADMOPs in fresh medium at 37 °C under aerobic or PDT‐induced hypoxic conditions. Cells incubated only in cell culture medium were prepared as untreated controls. The medium was aspirated after 24 h or 48 h of incubation, and the cells were washed twice with PBS to remove noninternalized probes. Cell viability was expressed as follows: Cell viability = (OD_sample_ − OD_blank_)/(OD_control_ − OD_blank_). The OD_sample_ and OD_control_ were the absorbance values of the treated cells (as indicated) and the untreated control cells (without nanoparticles), respectively. The OD_blank_ was the absorbance of CCK8 reagent itself at 450 nm.

*Synergistic Antitumor Efficacy of ZnPC‐ADMOPs In Vivo*: Male BALB/c‐nude mice (five weeks old) were purchased from China Wushi, Inc. (Shanghai, China). All animal procedures were approved by the Animal Ethics Committee of Fujian Medical University. Tumor‐bearing mice were prepared by subcutaneously injecting a suspension of HepG2 cells (5 × 10^6^ cells) in sterilized 1 × PBS buffer. When the tumor size reached ≈5–7 mm, the mice were divided into five groups: (i) sterilized PBS with combined laser irradiation under 670 nm (0.1 W cm^−2^) for 10 min (*n* = 4); (ii) free AQ4N without laser irradiation (*n* = 4); (iii) free DOX without laser irradiation (*n* = 4); (iv) ADMOPs without laser irradiation (*n* = 4); and (vi) ZnPC‐ADMOPs combined with laser irradiation under 670 nm (0.1 W cm^−2^) for 10 min (*n* = 4). Then, 200 µL AQ4N (23.4 µg mL^−1^), DOX (56.3 µg mL^−1^), ADMOPs (AQ4N, 23.4 µg mL^−1^; DOX, 56.3 µg mL^−1^) or ZnPC‐ADMOPs (AQ4N, 23.4 µg mL^−1^; DOX, 56.3 µg mL^−1^; ZnPC, 28.6 µg mL^−1^;) was intravenously injected into the mice of each respective group.

For the ex vivo fluorescence imaging experiments, HepG2‐bearing nude mice were intravenously injected with free DOX, free AQ4N, ADMOPs or ZnPC‐ADMOPs and then sacrificed by cervical dislocation 4 h after injection. After anatomization, the dissected organs, including the tumor, heart, liver, spleen, lungs, and kidneys were imaged with a ChemiDoc MP Imaging System (Bio‐Rad). The concentrations of Cu and Zn in these organs and tumors were quantified by ICP‐MS. For synergistic antitumoral efficacy analysis, irradiation was conducted 4 h after injection. The therapeutic effects were evaluated by monitoring the tumor volume and body weight change in each group every 2 d for up to 12 d. The tumor size was measured using calipers every other day after the treatment. The tumor volume (*V*) was calculated using the following equation

(1) $$V={\mathrm{AB}}^{2}/2$$

where *A* and *B* are the long and short diameters (mm) of the tumor, respectively.

*Histological Examination and Long‐Term Toxicity Assessment*: To examine the histological changes in the tumors after treatment, the tumor‐bearing mice in each group were sacrificed at 14 d, and the tumors were collected, weighed, and stained with H&E and Ki67 for histopathological evaluation. Immunohistochemical staining of tumor slices was performed with antibodies specific for HIF1α (1:800) (H1alpha67, Novus Biologicals, Littleton, CO or 54, BD Bioscience) and hematoxylin (to stain nuclei) of ZnPC‐ADMOP‐injected mice with or without 670 nm laser irradiation (0.1 W cm^−2^, 10 min) 1 h after injection. The long‐term systematic toxicity assessment was performed as follows: the ADMOP‐ and ZnPC‐ADMOP‐treated mice were sacrificed 14 d after treatment, and the major organs (heart, liver, spleen, lungs, and kidneys) of the mice were collected, fixed in 4% neutral formaldehyde, embedded in paraffin, stained with H&E, and observed under a Zeiss microscope (Axio Lab.A1).

*Statistical Analysis*: Statistical analysis of the data was performed using one‐way analysis of variance (ANOVA) or two‐tail paired Student's *t*‐tests (*p < 0.05, **p < 0.01, ***p < 0.001). All data are shown as the means ± SD of at least three experiments.

## Conflict of Interest

The authors declare no conflict of interest.

## Supporting information

Supplementary
