# Supplementary material for: Chemotherapeutic Drug Based Metal–Organic Particles for Microvesicle‐Mediated Deep Penetration and Programmable pH/NIR/Hypoxia Activated Cancer Photochemotherapy
Source: Adv Sci (Weinh). 2018 Jan 3;5(2):1700648. doi: 10.1002/advs.201700648 (PMC5827097; doi:10.1002/advs.201700648)
Supplement: Supplementary file 1 — Supplementary [file ADVS-5-1700648-s001.pdf]

Copyright WILEY-VCH Verlag GmbH & Co. KGaA, 69469 Weinheim, Germany, 2018.

Supporting Information

for *Adv. Sci.,* DOI: 10.1002/advs.201700648

Chemotherapeutic Drug Based Metal–Organic Particles for Microvesicle-Mediated Deep Penetration and Programmable pH/NIR/Hypoxia Activated Cancer Photochemotherapy

Da Zhang, Ming Wu, Zhixiong Cai, Naishun Liao, Kun Ke, Hongzhi Liu, Ming Li, Gang Liu, Huanghao Yang, Xiaolong Liu, and Jingfeng Liu**

Supporting Information

Chemotherapeutic drug based metal-organic particles for microvesicle-mediated deep penetration and pH/ NIR/hypoxia-programmable activated cancer photo-chemotherapy

Da Zhang[a,b], Ming Wu[a,b], Zhixiong Cai[a,b], Nai shunliao[a,b], Kun Ke [a], Hongzhi Liu[a], Ming Li[a], Gang Liu[e], Huanghao Yang[d], Xiaolong Liu[a,b], Jingfeng Liu[a,b,c]**

Figure S1

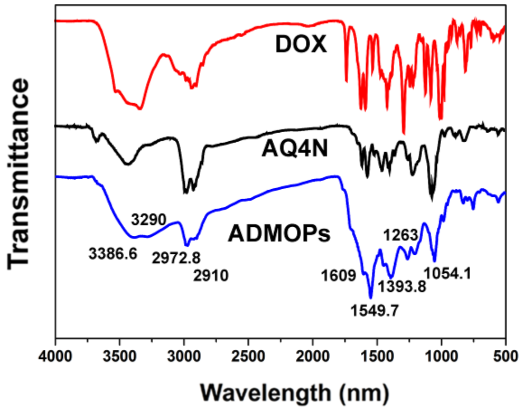

Figure S1. FT-IR spectra of AQ4N, DOX and ADMOPs.

Figure S2

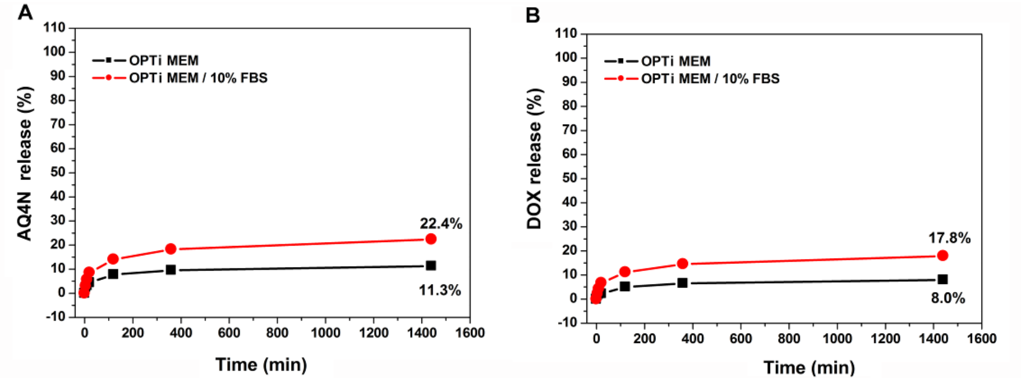

Figure S2. In vitro DOX or AQ4N release kinetics from ADMOPs in opti-MEM with or without 10% FBS at 37 °C.

Figure S3..

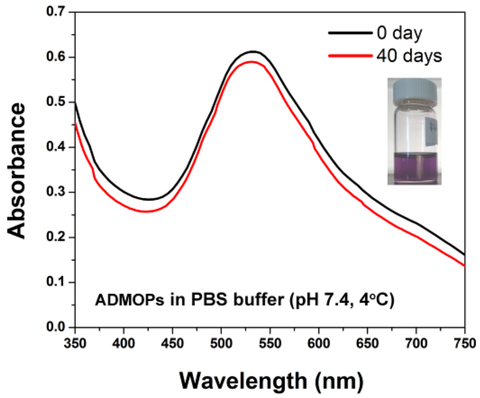

Figure S3. The stability of ADMOPs in PBS buffer (pH 7.4) over 40days.

Figure S4.

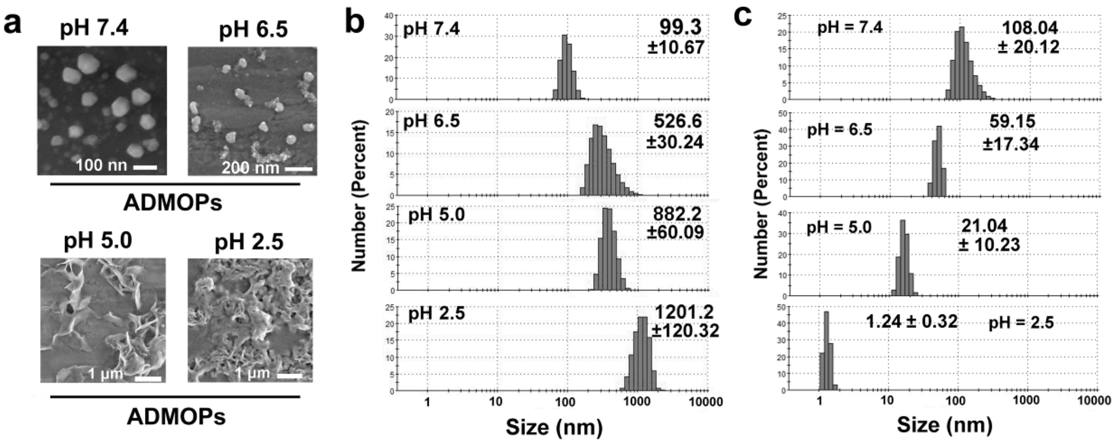

Figure S4. (a) SEM results of ADMOPs under different pH values over 24hrs. (b) DLS results of ADMOPs in different pH conditions over 24hrs. (c) DLS results of the supernatant obtained from centrifugation of the corresponding ADMOPs in (b).

Figure S5

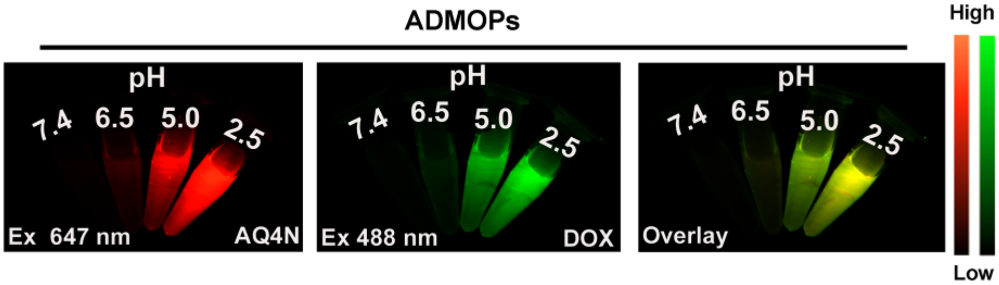

Figure S5. The fluorescence images of ADMOPs at different pH conditions (2.5~ 7.4).

Figure S6

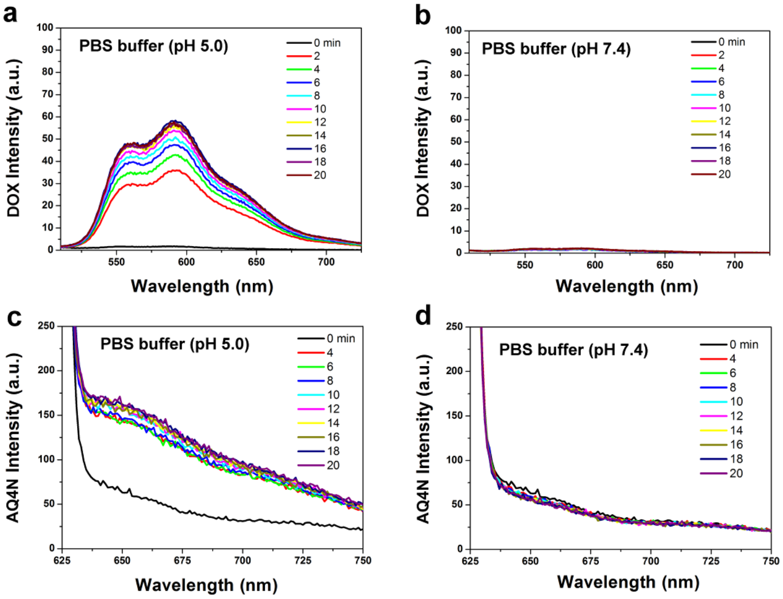

Figure S6. Fluorescence emission spectrum of ADMOPs (excitation at 488 nm for DOX or 625 nm for AQ4N) in different pH conditions as indicated (pH 5.0 and 7.4), respectively.

Figure S7

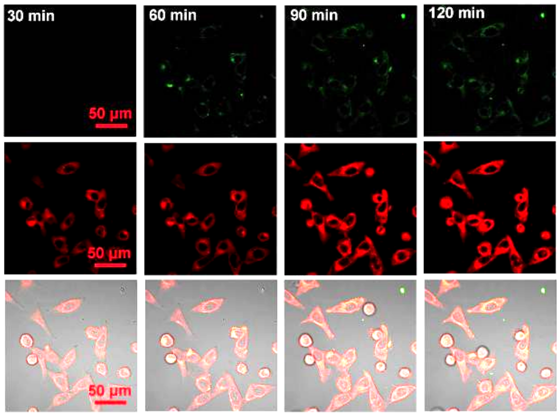

Figure S7. Confocal images of HepG2 cells incubated with ADMOPs for 30~120 min (scale bar = 50 μm).

Figure S8

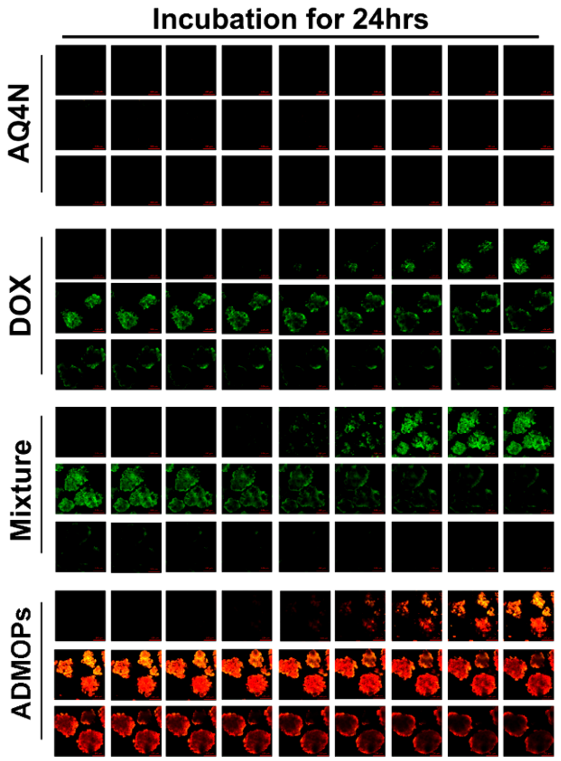

Figure S8. Z-stack images of 3D tumor spheroids that incubated with DOX alone, AQ4N alone, mixture of DOX/AQ4N, and ADMOPs for 24hrs. The images were taken every ~3.3 mm section from the top to bottom of intact 3D tumor spheroids, scale bar = 100 μm.

Figure S9

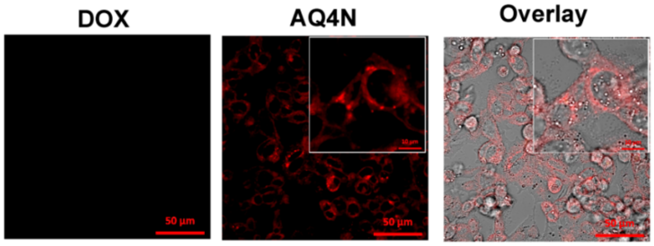

Figure S9. Confocal images of HepG2 cells treated with the supernatant drived from ADMOPs treated cells.

Figure S10

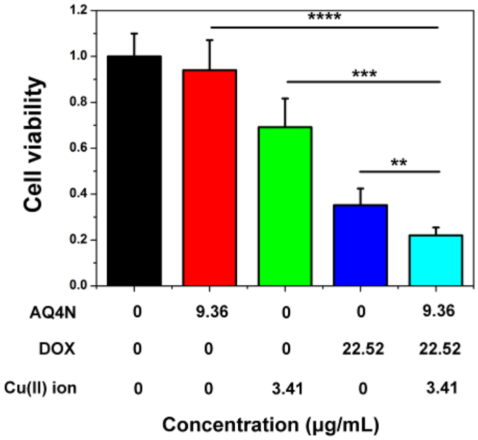

Figure S10. Cell viability of HepG2 cells treated with free DOX, AQ4N, Cu(II) ion and ADMOPs for 48hrs in aerobic condition (n = 5).

Figure S11

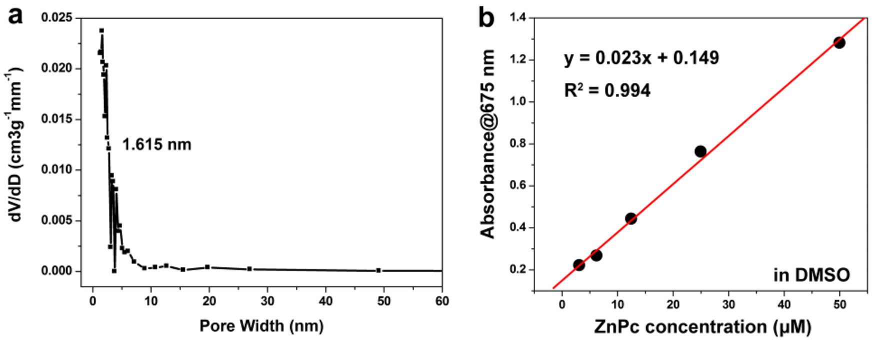

Figure S11. (a) The pore size distribution profile of ADMOPs. (b) The linear fit of the absorbance of various concentration of ZnPC from 3.125 to 50 μM in DMSO.

Figure S12

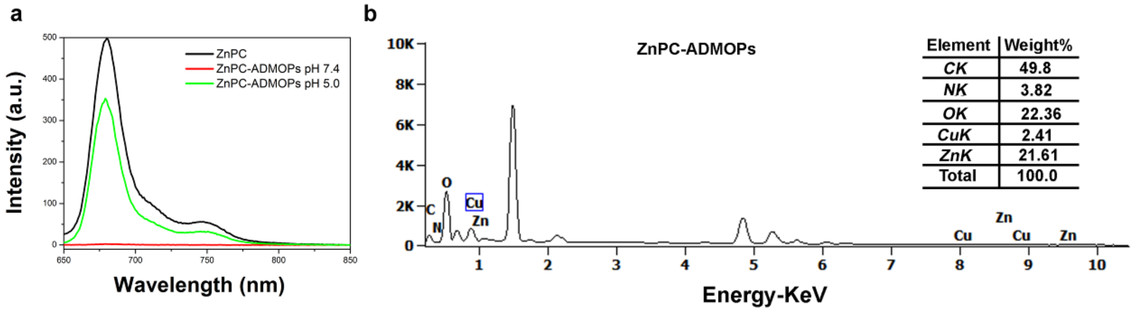

Figure S12. (a) Fluorescence spectra of ZnPC (DMSO) and ZnPC-ADMOPs in PBS buffer with different pH values; (b) Energy dispersive x-ray spectroscopy of ZnPC-ADMOPs.

Figure S13

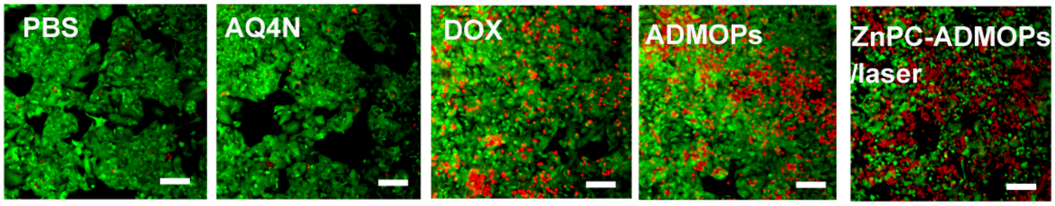

Figure S13. Fluorescence images of the live/dead viability toxicity kit stained HepG2 cells under the following conditions (all in aerobic conditions): HepG2 cells without treatment; HepG2 cells incubated with AQ4N; HepG2 cells incubated with DOX; HepG2 cells incubated with ADMOPs; HepG2 cells incubated with Zn-ADMOPs under the 670 nm laser; scale bar = 50 μm.

Figure S14

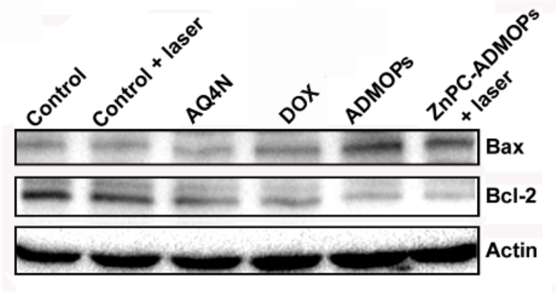

Figure S14. Western blot analysis of HepG2 cells treated with free AQ4N, DOX, ADMOPs and ZnPC-ADMOPs with 670 nm laser irradiation (0.1 W/cm^2, 10 min).

Figure S15

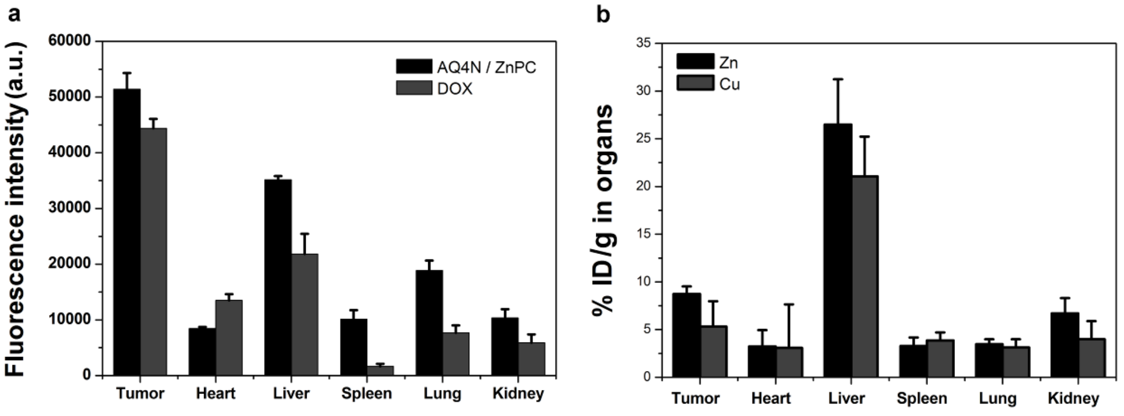

Figure S15. Organ distribution of ZnPC-ADMOPs after intravenous injection of ZnPC-ADMOPs in tumors-bearing mice at 4hrs of post-injection (n = 3), which was determined by fluorescence imaging (a) and by ICP-MS anaylsis (b).

Figure S16

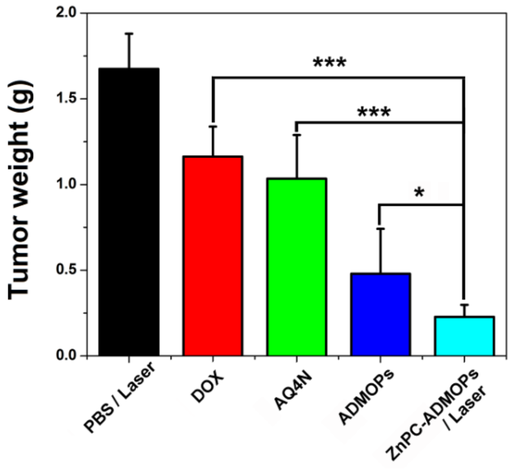

Figure S16. The average tumor weight of PBS treatment with laser irradiation, free DOX alone, free AQ4N alone, ADMOPs and ZnPC-ADMOPs with laser irradiation at the 14 days (n=4). The statistical analysis was performed with the two-tailed paired Student's T-test, *p<0.05, ***p<0.001. (670 nm, 0.1 W/cm^2)

Figure S17

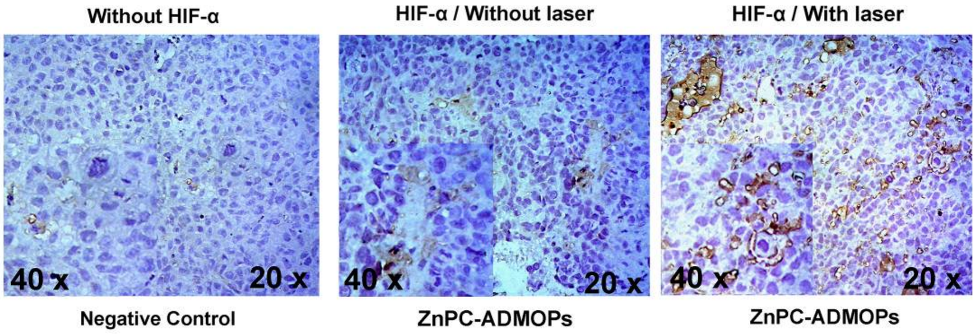

Figure S17. Ex vivo immunohistochemical staining of tumor slices collected from ZnPC-ADMOPs treated mice with or without 670 nm laser irradiation (0.1 W/cm^2, 10 min) after 1hrs of injection.

Figure S18

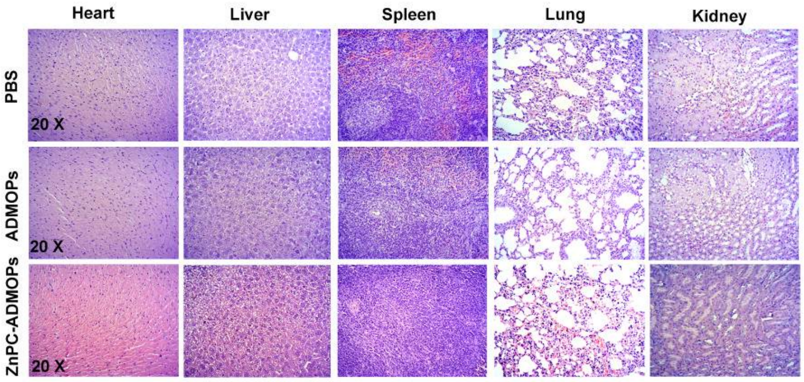

Figure S18. The pathological changes of main organs evaluated by H&E staining which were acquired at 14 days after intravenous injection of PBS, ADMOPs and ZnPC-ADMOPs. No noticeable pathological changes were observed in these organs.
